# Antioxidant, anti-apoptotic, and protective effects of myricitrin and its solid lipid nanoparticle on streptozotocin-nicotinamide-induced diabetic nephropathy in type 2 diabetic male mice

**DOI:** 10.22038/IJBMS.2019.13989

**Published:** 2019-12

**Authors:** Akram Ahangarpour, Ali Akbar Oroojan, Layasadat Khorsandi, Maryam Kouchak, Mohammad Badavi

**Affiliations:** 1 Department of Physiology, Faculty of Medicine, Diabetes Research Center, Health Research Institute, Ahvaz Jundishapur University of Medical Sciences, Ahvaz, Iran; 2 Department of Physiology, Faculty of Medicine, Cellular and Molecular Research Center, Student Research Committee of Ahvaz Jundishapur University of Medical Sciences, Ahvaz, Iran; 3 Department of Physiology, Faculty of Medicine, Dezful University of Medical Sciences, Dezful, Iran; 4 Department of Anatomical Sciences, Faculty of Medicine, Cellular and Molecular Research Center, Ahvaz Jundishapur University of Medical Sciences, Ahvaz, Iran; 5 Department of Pharmaceutics, Faculty of Pharmacy, Nanotechnology Research Center, Ahvaz Jundishapur University of Medical Sciences, Ahvaz, Iran; 6 Department of Physiology, Faculty of Medicine, Physiology Research Center, Ahvaz Jundishapur University of Medical Sciences, Ahvaz, Iran

**Keywords:** Antioxidant, Anti-apoptotic, Diabetic nephropathy, Mice, Myricitrin, Solid lipid nanoparticle

## Abstract

**Objective(s):**

The present study evaluates the protective effects of myricitrin and its solid lipid nanoparticle (SLN) on diabetic nephropathy (DN) induced by streptozotocin-nicotinamide (STZ-NA) in mice.

**Materials and Methods:**

In this experimental study, 108 adult male NMRI mice were divided into 9 groups: control, vehicle, diabetes, diabetes + myricitrin 1, 3, and 10 mg/kg and, diabetes + SLN containing myricitrin 1, 3, and 10 mg/kg. After the experimental period, the plasma and tissue samples were collected for experimental, histopathological, real-time PCR and apoptosis assessments.

**Results:**

Total antioxidant capacity, catalase, glomerular filtration rate, plasma level of albumin, urine (BUN) and, creatinine (Cr) levels decreased, and the kidney weight, intake/output, malondialdehyde, plasma level of BUN and Cr, urine level of sodium, potassium, albumin and glucose, fractional excretions of sodium and potassium, transforming growth factor-β (TGF-β) and nuclear factor kappa B (NF-κB) gene expression, red blood cell accumulation and infiltration of inflammatory cells, and kidney apoptosis increased in untreated diabetic mice compared to the control group, and administration of myricitrin and its SLN recovered all of these changes.

**Conclusion:**

Ultimately, myricitrin and its SLN administration improved DN changes by reducing oxidative stress and increasing antioxidant enzymes level, and these effects were more prominent in the SLN-administered mice.

## Introduction

Diabetic nephropathy (DN), known as a kidney progressive disease is characterized by persistent albuminuria, progressive decrease in glomerular filtration rate (GFR) and increases in plasma level of creatinine (Cr). This disease occurs in 45 % of diabetic patients (1). The renal hemodynamic alteration can activate oxidative stress via increasing lipid peroxidation and reactive oxygen species (ROS) and decreasing antioxidant defense in DN. Moreover, the release of pro-inflammatory agents such as nuclear factor kappa B (NF-κB) and transforming growth factor-β (TGF-β) are responsible for the development and maintenance of DN (2, 3). Among the antioxidants, phenolic compounds have a main role in the treatment of type 2 diabetes mellitus (T2DM) and DN by reducing free radicals (4). Myricitrin is an important flavonol glycoside derived from the root bark of *Myrica cerifera *(5). This plant-derived flavonol glycoside has considerable hypoglycemic, antioxidant, anti-nociceptive, anti-inflammatory, and anti-apoptotic activities (6). Oral bioavailability of the polyphenols is relatively low because they are large and polar that cannot pass through the cell membranes (7). Solid lipid nanoparticles (SLNs), as a novel nano drug delivery system, have several advantages such as nontoxic side effects, sustained and controlled release and the stability of unstable substances, which enhance the oral bioavailability of flavonoids (8). Streptozotocin-nicotinamide (STZ-NA)-induced T2DM that results in DN, renal dysfunction and, glomerular injury by increasing free radicals diminished antioxidant defense and Cr clearance (9). However, STZ increased lipid peroxidation due to high blood glucose levels in the kidney, but this compound does not have nephrotoxicity, and all changes in the kidney function can be attributed to the altered metabolism in diabetes (4). Moreover, a single dose of STZ could produce renal tumors, and increased DNA methylation by STZ in the liver and kidney can destroy normal metabolism in these organs. This event makes STZ-induced type 1 diabetes as an unsuitable model for long-term effects of diabetes on the kidney, while the STZ-NA model of type 2 diabetes has been reported to be a suitable model for studies focused on diabetes complications such as DN (10). So, based on the effect of T2DM on inducing oxidative stress in the kidney that leads to DN and, the antioxidant effects and poor bioavailability of myricitrin for improvement of DN, the present study aims to evaluate the kidney protective effects of myricitrin and its SLN on STZ-NA-induced DN in type 2 diabetic male mice.

## Materials and Methods

***Chemicals and experimental kits***

Myricitrin (Purity 98 %) (AvaChem Scientific, USA), Citrate buffer, Tween 80 (Sinopharm, China), Streptozotocin (Solar bio, South Korea), Nicotinamide, Hematoxylin, Eosin, (Sigma, USA), Ketamine/Xylazine (Alfasan, Netherland), phosphate-buffered saline (PBS) (Pharmaceutical Technology Development Center of Ahvaz Jundishapur University of Medical Sciences (AJUMS), Iran), malondiadehyde (MDA), total oxidant capacity (TAC), catalase (CAT) (Zellbio, Germany), superoxide dismutase (SOD) (Randox Laboratories Ltd. United Kingdom) assay kits, blood urea nitrogen (BUN), Cr, albumin, glucose assay kits (Pars Azmoon, Iran), RNeasy Mini Kit (Qiagen, Valencia, CA), cDNA Synthesis Kit, Sybergreen (Takara, Japan), TGF-β and NF-κb, and glyceraldehyde-3-phosphate dehydrogenase (GAPDH) primers (Microsynth, Switzerland), TUNEL assay kit (Roche Applied Science, Germany), Proteinase K (Invitrogen, Thermo Fisher Scientific, Germany).

***Preparation of myricitrin SLNs and its characterization***

SLNs of myricitrin were prepared according to the cold homogenization method explained in our previous study. Also, the particle characteristics such as size, zeta potential, drug loading (DL %) and encapsulation efficiency (EE %) were assessed in that study (11).

***Animals***

In the present experimental study, 108 adult male Naval Medical Research Institute (NMRI) mice (25-30 g) were obtained from the Ahvaz Jundishapur University of Medical Sciences (AJUMS) animal facility and, treated in accordance with the principles and guidelines on animal care of AJUMS as reviewed by an ethics committee (IR.AJUMS.REC.1395.136) and kept at a 20 °C ± 4 °C temperature with a 12 hr / 12 hr light and dark cycle. They received tap water and commercial chow *ad libitum*. After one-week acclimatization of mice, T2DM induced by intraperitoneal injection of STZ (65 mg/kg) and NA (120 mg/kg) (dissolved in a citrate buffer (pH: 4.5) and normal saline, respectively) with 15 min interval. Mice with fasting blood glucose above 200 mg/dl were considered as diabetic and entered the study at 3 days after STZ-NA administration (12). Therefore, the animals were divided into 9 groups (n = 12) as the following: 

**Group I:** Control

**Group II:** Vehicle (received STZ-NA and myricitrin (Tween-80 3 % in addition to Saline 97 %) solvents)

**Group III:** Diabetes

**Groups IV, V and, VI:** Diabetes in addition to administration of Myricitrin 1, 3, and 10 mg/kg, respectively.

**Groups VII, VIII and, IX:** Diabetes in addition to administration of SLN containing myricitrin 1, 3, and 10 mg/kg, respectively (13). 

The oral drug administration period was 28 days to protect the mice from DN. At the end of the experiment, mice were individually housed in metabolic cages for 24 hr intake / output assessment. Six hours after myricitrin and SLN containing myricitrin utilization, blood samples were collected by cardiac puncture from the 12 hr fasted and Ketamine / Xylazine (70 / 10 mg/kg) injected anesthetized mice. Then, plasma samples were prepared by blood samples centrifuging at 3500 rpm for 20 min. Following that, the kidney of all animals was separated for histological, gene expression, and apoptosis assessment. The plasma and kidney samples were maintained at −80 °C until the laboratory measurements were performed (14). 

***Experimental measurements***

The kidney homogenate was prepared using Sharma and Singh method. In brief, the separated kidney slab on the dry ice pack, homogenized in 1/5 (w/v) PBS (pH: 7.4) with a Teflon homogenizer, and centrifuged at 2000 rpm for 10 min. Finally, the supernatant was used to measure the levels of lipid peroxidation (MDA) and antioxidant defense variables (TAC, CAT and, SOD) by specific commercial kits (15).

Plasma and urine level of the BUN, Cr, albumin, sodium (Na), potassium (K) and, urine level of glucose were measured by using an Autoanalyzer (BT3000, Italy) electrolyte analyzer devices and biochemical assay kits (Pars Azmoon, Iran). Also, GFR, and fractional excretion of sodium and potassium (FE Na, and FE K) were assessed by the following formula: GFR= Urine Cr × Urine volume / Plasma Cr; FE Na= (Urine Na × Plasma Cr / Plasma Na × Urine Cr) × 100; FE K= (Urine K × Plasma Cr / Plasma K × Urine Cr) × 100 (16).

***Gene expression assessment***

Real-time PCR method was used for gene expression measurement in the present study. In brief, total RNA and cDNA were purified and synthesized from the kidney by the RNeasy mini kit and Reverse Transcriptase kit, respectively according to their instructions (17). Real-time PCR was performed with SYBR Green Master Mix in ABI step one plus instrument (Thermofisher, USA) , and was prepared in triplicate. The expression levels of the TGF-β and NF-κb were normalized against the endogenous reference gene GAPDH (as a housekeeping and suitable endogenous internal control for gene expression in kidney studies) (18) and calculated using a comparative CT method (2^-ΔΔCT^). The sequences of forward and reverse primer for TGF-β, NF-κb, and GAPDH genes are presented in Table 1 (17).

***Histopathological and apoptosis assessments***

Histopathological assessment was performed by fixation of the kidney tissue in formalin solution (10 %) and hematoxylin and eosin (H&E) staining method. Then, seven microscopic slides of tissue sections (5 to 7 µm) were assessed and read using blind method. Apoptosis assessment was carried out by TUNEL staining according to the labeling of DNA strand breaks by administration of *In Situ* Cell Death Detection Kit, POD, and the dark brown stained nucleus was included as dead cells. The index of apoptosis was measured in 3 randomly slides/animal and ten fields for each slide as a percentage of TUNEL-positive cells (19).

**Table 1:** The forward and reverse primer sequences

**Genes**	**Forward primer**	**Reverse primer**
**TGF-β**	5′-GCCTGGACACACAGTACAGC-3′	5′-TTGCAGGAGCGCACAATCAT-3′
**NF-κB**	5′-GAGCTGCTGCATTTCCAGGT-3′	5′-AGGCCTGTTCCCTCTGACTC-3′
**GAPDH**	5′-ACCCAGAAGACTGTGGATGG-3′	5′-TTCTAGACGGCAGGTCAGGT-3′

NF-κB: Nuclear factor kappa B; TGF-β: Transforming growth factor-β; GAPDH: Glyceraldehyde-3-phosphate dehydrogenase

**Figure 1:**
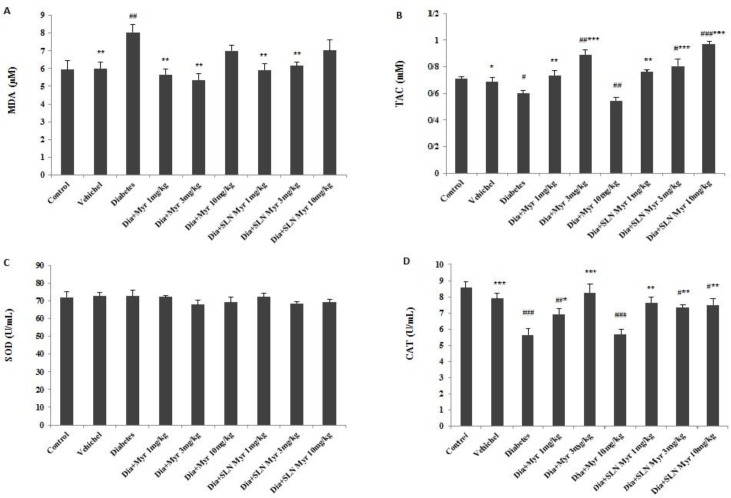
The effects of myricitrin and its SLN on lipid peroxidation and antioxidant enzyme level of the kidney. Data are presented as mean±SEM; n=12; #*P*<0.05, ##*P*<0.01 and ###*P*<0.001 compared to the control, *P<0.05, **P<0.01 and ***P<0.001 compared to the diabetes group. (Dia: Diabetes), (Myr: Myricitrin), (SLN: Solid lipid nanoparticle), (SOD: Superoxide dismutase), (CAT: Catalase), (MDA: Malondialdehyde), (TAC: Total antioxidant capacity). (A) MDA, (B) TAC, (C) SOD, (D) CAT

**Table 2:** Effect of myricitrin and its SLN on intake, output, antioxidant, lipid peroxidation, and weight of the kidney

**Groups**	**Kidney weight** **(g)**	**Water intake** **(ml)**	**Food intake** **(g)**	**Urine volume** **(ml)**
**Control**	0.184±0.007	9.05±0.37	5.31±0.18	0.666±0.016
**Vehicle**	0.182±0.004^**^	9.40±0.36^*^	5.37±0.19^**^	0.660±0.018^**^
**Diabetes**	0.209±0.007^##^	10.58±0.31^#^	6.66±0.48^##^	0.780±0.020^##^
**Diabetes ** **+ ** **myricitrin 1 mg/kg**	0.183±0.006^**^	9.10±0.42^*^	5.45±0.31^**^	0.687±0.023^**^
**Diabetes ** **+ ** **myricitrin 3 mg/kg**	0.185±0.005^**^	8.77±0.43^*^	5.78±0.13^**^	0.700±0.018^**^
**Diabetes ** **+ ** **myricitrin 10 mg/kg**	0.183±0.009^**^	8.25±0.32^**^	5.68±0.17^**^	0.687±0.024^**^
**Diabetes + myricitrin SLN 1 mg/kg**	0.180±0.006^**^	8.50±0.27^**^	5.13±0.13^***^	0.650±0.028^**^
**Diabetes + myricitrin SLN 3 mg/kg**	0.182±0.004^*^	8.40±0.41^**^	5.01±0.21^***^	0.688±0.023^**^
**Diabetes + myricitrin SLN 10 mg/kg**	0.187±0.009^**^	8.22±0.40^**^	4.96±0.33^***^	0.640±0.018^***^

Data are presented as mean±SEM; n=12; #*P*<0.05, ##*P*<0.01 and ###*P*<0.001 compared to the control, *P<0.05, **P<0.01, and ***P<0.001 compared to the diabetes group. SLN: Solid lipid nanoparticle

**Table 3:** Effect of myricitrin and its SLN on GFR, urine glucose, plasma and urine level of BUN, Cr and, Alb

**Groups**	**Plasma BUN** **(mg/dl)**	**Plasma Cr** **(mg/dl)**	**Plasma Alb** **(g/dl)**	**GFR** **(ml/min)**
**Control**	42.43±0.68	0.241±0.005	3.11±0.05	95.34±6.42
**Vehicle**	42.20±0.65^***^	0.261±0.005^***^	3.12±0.06^***^	84.04±7.99^**^
**Diabetes**	58.67±0.71^###^	0.322±0.008^###^	2.64±0.03^###^	68.25±2.14^###^
**Diabetes ** **+ ** **myricitrin 1 mg/kg**	54.02±0.43^###**^	0.297±0.006^###*^	2.73±0.01^###^	79.68±4.01^#*^
**Diabetes ** **+ ** **myricitrin 3 mg/kg**	47.71±0.97^##***^	0.292±0.008^###*^	2.78±0.04^###*^	81.11±2.99^*^
**Diabetes ** **+ ** **myricitrin 10 mg/kg**	45.80±1.22^#***^	0.341±0.004^###^	2.71±0.05^###^	51.03±2.02^###*^
**Diabetes + myricitrin SLNs 1 mg/kg**	48.08±1.60^##***^	0.252±0.013^***^	2.63±0.03^###^	83.08±4.30^*^
**Diabetes + myricitrin SLNs 3 mg/kg**	40.82±0.88^***^	0.233±0.008^***^	2.78±0.04^###*^	98.85±5.95^***^
**Diabetes + myricitrin SLNs 10 mg/kg**	39.43±0.32^***^	0.310±0.004^###^	2.75±0.04^###^	53.95±2.66^###*^

Data are presented as mean±SEM; n=12; #*P*<0.05, ##*P*<0.01 and ###*P*<0.001 compared to the control, *P<0.05, **P<0.01, and ***P<0.001 compared to the diabetes group. SLN: Solid lipid nanoparticle, GFR: Glomerular filtration rate, Cr: Creatinine, BUN: Blood urea nitrogen, Alb: Albumin

**Figure 2:**
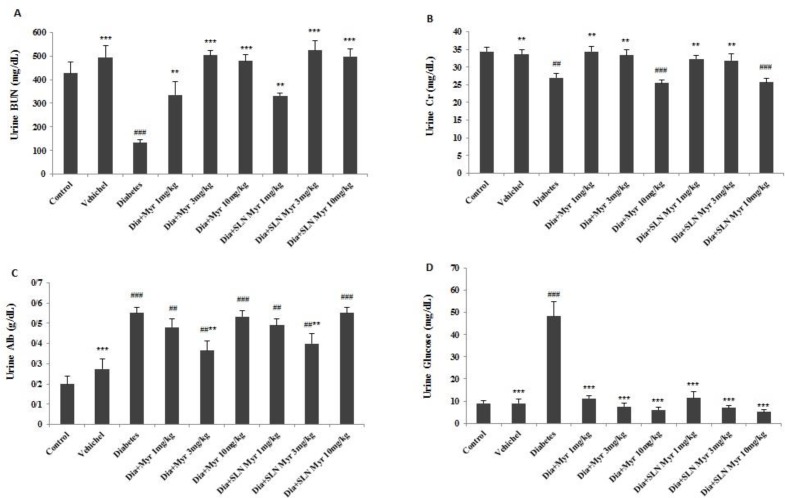
The effects of myricitrin and its SLN on urine level of BUN, Cr, albumin and, glucose. Data are presented as mean±SEM; n=12; ##*P*<0.01 and ###*P*<0.001 compared to the control, **P<0.01 and ***P<0.001 compared to the diabetes group. (Dia: Diabetes), (Myr: Myricitrin), (SLN: Solid lipid nanoparticle), (Cr: Creatinine), (BUN: Blood urea nitrogen). (A) Urine BUN, (B) Urine Cr, (C) Urine Albumin, (D) Urine glucose

**Table 4:** Effect of myricitrin and its SLN on plasma and urine levels of Na and K, FE Na, FE K, and relative mRNA expression of TGF-β and NF-κB in the kidney

**Groups**	**Plasma Na** **(mEq/l)**	**Plasma K** **(mEq/l)**	**Urine Na** **(mEq/l)**	**Urine K** **(mEq/l)**	**FE Na** **(%)**	**FE K** **(%)**
**Control**	171.25±1.65	5.52±0.28	136.33±11.86	75.50±5.90	0.525±0.016	9.54±1.07
**Vehicle**	170.40±2.63	5.28±0.37	138.50±12.58^**^	71.66±6.98^**^	0.602±0.100^***^	10.09±1.98^***^
**Diabetes**	173.33±2.02	5.10±0.28	185.33±12.97^##^	105.50±9.13^##^	1.277±0.138^###^	24.60±1.10^###^
**Diabetes ** **+ ** **myricitrin 1 mg/kg**	174.00±1.68	5.25±0.12	170.50±10.50^#^	87.33±5.84^*^	0.847±0.023^#**^	14.38±0.89^#***^
**Diabetes ** **+ ** **myricitrin 3 mg/kg**	173.66±6.76	5.10±0.23	136.33±7.02^**^	85.50±6.78^*^	0.687±0.100^***^	14.62±2.5^#***^
**Diabetes ** **+ ** **myricitrin 10 mg/kg**	168.50±1.04	5.25±0.11	139.33±15.50^**^	70.25±2.21^***^	1.104±0.098^###^	17.87±0.83^##**^
**Diabetes + myricitrin SLNs 1 mg/kg**	180.25±5.75	5.55±0.17	166.33±10.96^#^	82.50±4.52^*^	0.720±0.069^***^	11.63±1.60^***^
**Diabetes + myricitrin SLNs 3 mg/kg**	171.75±3.37	5.70±0.10	125.33±6.17^**^	79.25±6.15^**^	0.535±0.045^***^	10.02±1.05^***^
**Diabetes + myricitrin SLNs 10 mg/kg**	169.25±3.94	5.47±0.08	135.50±13.02^**^	69.00±4.72^***^	0.961±0.100^##*^	15.18±1.70^##***^

Data are presented as mean±SEM; n=12; #*P*<0.05, ##*P*<0.01 and ###*P*<0.001 compared to the control, *P<0.05, **P<0.01, and ***P<0.001 compared to the diabetes group. SLN: Solid lipid nanoparticle, FE Na: Fractional excretion of sodium, FE K: Fractional excretion of potassium, NF-κB: Nuclear factor kappa B, TGF-β: Transforming growth factor-β

**Figure 4:**
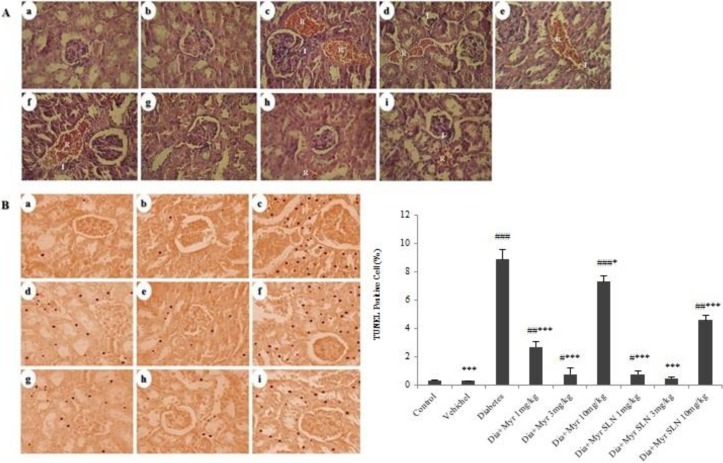
The effect of myricitrin and its SLN on the kidney histopathology and apoptosis. (A) Kidney histopathology (Hematoxylin & Eosin; ×40 magnification). (B) Kidney apoptosis (TUNEL; ×40 magnification). (a) Control. (b) Vehicle. (c) Diabetes. (d) Diabetes + myricitrin 1 mg/kg. (e) Diabetes + myricitrin 3 mg/kg. (f) Diabetes + myricitrin 10 mg/kg. (g) Diabetes + SLN containing myricitrin 1 mg/kg. (h) Diabetes + SLN containing myricitrin 3 mg/kg. (i) Diabetes + SLN containing myricitrin 10 mg/kg. Data are presented as mean±SEM; n=3; #*P*<0.05, ##*P*<0.01 and ###*P*<0.001 compared to the control, *P<0.05 and ***P<0.001 compared to the diabetes group. (Dia: Diabetes), (Myr: Myricitrin), (SLN: Solid lipid nanoparticle)

***Statistics***

The obtained results were statistically analyzed by SPSS software (version 16) with one-way analysis of variance (ANOVA) and *post hoc* least significant difference (LSD) tests. Moreover, the data were represented as the mean±standard error of the mean (SEM), and the statistically significant differences were considered at *P*<0.05.

## Results

***The role of myricitrin and its SLN on kidney weight and intake/output***

As shown in Table 2, the kidney weight increased in diabetes (*P*<0.01) compared to the control and decreased in the groups II (*P*<0.01), IX (*P*<0.05), and others (*P*<0.01) versus diabetes group. The water and food intake showed a significant increase in diabetic mice when compared to control (*P*<0.05 and *P*<0.01, respectively). The water intake decreased in groups IV, V (*P*<0.05), and others (*P*<0.01) compared to the diabetes group. Food intake assessment showed a similar effect in the groups II, IV, V, VI (*P*<0.01), VII, VIII, and IX (*P*<0.001) compared to diabetes group. The urinary volume of untreated diabetic mice increased significantly (*P*<0.01). Further, the urine output decreased in groups IX (*P*<0.001) and others (*P*<0.01) versus diabetes group.

***Lipid peroxidation and antioxidant enzymes level in the kidney***

MDA increased in diabetes when compared to control (*P<*0.01). This factor of lipid peroxidation reduced in the groups II (*P<*0.05), IV, V (*P<*0.01), VI, and VII (*P<*0.01) versus diabetes group (Figure 1A). The level of TAC decreased in diabetes (*P<*0.05) and group VI (*P<*0.01), and increased in groups V (*P<*0.01), VIII (*P<*0.05), and IX (*P<*0.001) in comparison with control. Further, this variable increased in groups II (*P<*0.05), IV (*P<*0.01), V (*P<*0.001), VII (*P<*0.01), VIII and IX (*P<*0.001) compared to untreated diabetic mice (Figure 1B). SOD measurement indicated no significant changes between all groups (Figure 1C). However, CAT level decreased in diabetes (*P<*0.001), groups IV (*P<*0.01), VI (*P<*0.001), VIII and IX (*P<*0.05) when compared to control, but this variable increased in groups II (*P<*0.001), IV (*P<*0.05), V (*P<*0.001), VII, VIII (*P<*0.01), and IX (*P<*0.01) versus diabetes group (Figure 1D). 

***The role of myricitrin and its SLN on GFR, urine glucose and plasma and urine levels of BUN, Cr, albumin, Na, K, FE Na, and, FE K***

As shown in Table 3, GFR decreased in diabetes and groups VI and IX compared to the control group (*P<*0.001). This variable increased in the groups II (*P<*0.01), VIII (*P<*0.001), and others (*P<*0.05) versus diabetes group. Plasma level of BUN increased in diabetes (*P<*0.001) and groups IV (*P<*0.001), V (*P<*0.01), VI (*P<*0.05), and VII (*P<*0.01) compared to the control. Also, this factor decreased in the groups II (*P<*0.001), IV (*P<*0.01), and others (*P<*0.001) compared to untreated diabetic mice. Plasma Cr level showed a significant increase in diabetes and groups IV, V, VI, and IX in comparison with the control group (*P<*0.001). This variable decreased in the groups II (*P<*0.001), IV, V (*P<*0.05), VIII, and IX (*P<*0.001) versus diabetes group. Plasma level of albumin reduced in all experimental groups except group II versus the control (*P<*0.001). Moreover, this factor increased in the groups II (*P<*0.001), V, and VIII (*P<*0.05) when compared to the diabetes group (Table 3). The BUN level of 24 hr urine decreased in untreated diabetic mice compared to the control group (*P<*0.001). Further, this variable increased in the groups IV, VII (*P<*0.01), and others (*P<*0.001) versus diabetes group (Figure 2A). The Cr level of 24 hr urine measurement revealed a significant decrease in diabetes (*P<*0.01) and groups VI and IX (*P<*0.001) compared to the control group. Also, this factor increased in the groups II, IV, V, VII, and VIII (*P<*0.01) when compared to diabetes group (Figure 2B). The 24 hr urine level of albumin increased in diabetes (*P<*0.001) and groups IV, V (*P<*0.01), VI (*P<*0.001), VII (*P<*0.01), VIII (*P<*0.01), and IX (*P<*0.001) versus the control group. Moreover, urine albumin level decreased in the groups II (*P<*0.001), V, and VIII (*P<*0.01) when compared to untreated diabetic mice (Figure 2C). The level of urine glucose increased in diabetes group versus control and decreased in other groups compared to diabetes group (*P<*0.001; Figure 2D). There were no significant differences in plasma level of Na and K between all groups. The 24 hr urine level of Na increased in diabetes and groups IV and VII (*P<*0.05) compared to the control. Also, this variable decreased in all groups (*P<*0.01) except IV and VII versus the diabetes group. The urine K level increased in diabetes compared to control (*P<*0.01). Also, this factor decreased in the groups II (*P<*0.01), IV, V (*P<*0.05), VI (*P<*0.001), VII (*P<*0.05), VIII (*P<*0.01), and IX (*P<*0.001) when compared to diabetes group. FE Na showed a remarkable increase in diabetes (*P<*0.001) and groups IV (*P<*0.05), VI (*P<*0.001) and, IX (*P<*0.01) versus to the control group. Further, this variable reduced in the groups II (*P<*0.001), IV (*P<*0.01), V (*P<*0.001), VII, VIII (P < 0.001) and, IX (*P<*0.05) compared to the diabetes. The results of FE K indicated a significant increase in diabetes (*P<*0.001) and groups IV, V (*P<*0.05), VI (*P<*0.01), and IX (*P<*0.01) in comparison with the control group. Moreover, FE K decreased in the groups VI (*P<*0.01) and others (*P<*0.001) when compared to the diabetes group (Table 4). 

***Effect of myricitrin and its SLN on relative mRNA expression of TGF-β and NF-*** ***κB*** ***in the kidney***

The gene expression of TGF-β increased in diabetes (*P<*0.001) and groups V (*P<*0.01), VI (*P<*0.001), VIII (*P*<0.05), and IX (*P*<0.001) compared to the control group. Also, TGF-β gene expression decreased in the groups II (*P*<0.001), IV (*P*<0.001), V (*P*<0.01), VII, and VIII (*P*<0.001), and increased in the group IX (*P*<0.001) when compared to the diabetes group (Figure 3A). NF-κb gene expression showed a remarkable increase in diabetes (*P*<0.001) and groups VIII (*P*<0.01) and IX (*P*<0.001) versus the control group. This variable decreased in the groups II (*P*<0.001), IV (*P*<0.001), V (*P*<0.01), VII (*P*<0.001), and VIII (*P*<0.001) compared to the diabetes group (Figure 3B).

***Effect of myricitrin and its SLN on the kidney histopathology and apoptosis***

The histopathological assessment showed an increase in red blood cell (RBC) accumulation and infiltration of inflammatory cells in diabetes group compared to the control group. These alterations decreased in the groups II and others treated groups versus diabetes group, and this decreasing effect was more obvious in the groups VII and VIII (Figure 4A). The results of kidney apoptosis revealed a significant increase in diabetes (*P*<0.001) and groups IV (*P*<0.01), V (*P*<0.05), VI (*P*<0.001), VII (*P*<0.05), and IX (*P*<0.01) when compared to the control. Furthermore, this variable decreased in the groups II (*P*<0.001), IV, V (*P*<0.001), VI (*P*<0.05), VII, VIII, and IX (*P*<0.001) compared to the diabetes group (Figure 4B).

## Discussion

The present study showed that polyphagia, polydipsia, and polyuria occurred in untreated diabetic mice, and myricitrin and its SLN administration improved these disorders. The present results indicated that the kidney weight increased in diabetic mice and administration of myricitrin and its SLN recovered this alteration. Consists with this result Sheela *et al.* demonstrated an increase in kidney weight of type 2 diabetic rats due to renal enlargement, hypertrophy, and hyperfunctioning, and treatment with silymarin as a plant-derived antioxidant improved kidney weight (20). When the capacity of the kidney decreases to absorb glucose, the sugar is filtered out via urination and produces hyperosmotic urination that leads to polyuria and loss of water and electrolyte. This event activates the thirst mechanism and polydipsia. Furthermore, glucosuria and tissue catabolism induce a lack of nourishment senses in the body and cause to the increase in appetite and polyphagia (21). Therefore, it can be suggested that STZ-NA-induced polyphagia, polydipsia, and polyuria along with T2DM via the above mechanism, and myricitrin and its SLN consumption improved them by their anti-diabetic properties.

T2DM increases ROS production and attenuates free radical scavenging molecules such as antioxidant enzymes. Free radicals induce basement membrane damage and lead to altering the membrane fluidity, ion transports and increased urinary albumin excretion (22). It seems that flavonoids have antioxidant or prooxidant properties depending on their dose of administration. Furthermore, the prooxidant effects of flavonoids can be useful, because, by imposing a mild degree of oxidative stress, the levels of TAC and antioxidant enzymes might be raised, which results in overall cytoprotection (23). Hence, present results indicated that T2DM model increased lipid peroxidation, and hydrogen peroxide and decreased TAC and CAT in the kidney. However, administration of low and moderate doses of myricitrin and its SLN recovered these changes, but a high dose of myricitrin or SLN containing myricitrin did not improve lipid peroxidation in diabetic mice, and it may be related to the administered concentration of this flavonoid glycoside, which acts as a prooxidant and does not induce antioxidant defense in myricitrin-administered mice or may induce a slight oxidative stress and increase TAC and CAT levels in myricitrin SLNs-treated mice.

Urinary albumin excretion, hypoalbuminemia, elevated BUN, serum Cr, urine glucose, and decreased GFR levels occurred in DN, which may be related to T2DM and formation of free radicals (24). The enhancement of urinary volume in T2DM disease leads to the loss of electrolytes and water and induces the imbalance of sodium and potassium levels in the body via osmotic dieresis and Na/K-ATPase pump dysfunction. Hyperglycemia-induced water movements out of the cells lead to reduced plasma level of Na, and the glucosuria-induced osmotic dieresis results in increasing the plasma Na level; hence, the interaction between these events causes to the normal plasma level of Na (25, 26). The level of FE Na and FE K depends on urine and plasma level of Cr, Na or K and tubular function. The increase in urine level of Na or K leads to the increased percentage of FE Na and FE K (27). Diabetes and renal oxidative stress increase and activate TGF-β and NF-κB that results in the secretion of inflammatory factors, and renal fibrosis, and decreasing urine BUN and Cr levels, which may also promote the progression of DN (28). Therefore, the results of the present study demonstrated that STZ-NA-induced T2DM cause to the DN symptoms through induction of oxidative stress, lipid peroxidation, decreasing antioxidant enzymes level and other mechanisms as mentioned above. Consists with the results of myricitrin and its SLN, as a flavonol glycoside, it was revealed that administration of flavonoids extracted from the leaves of *Murraya paniculata* ameliorated kidney dysfunctions of DN in diabetic rats through reducing oxidative stress and increasing antioxidant enzymes level (29). Moreover, SLN containing myricitrin is more potent than myricitrin in the improvement of BUN, urine Cr, GFR, Na, K, FE Na, FE K, TGF-β, and NF-κB gene expression. Furthermore, these effects were more evident in low and moderate doses of myricitrin and its SLN consumption, and high administered dose did not improve some of these variables such as plasma and urine level of Cr and albumin or TGF-β and NF-κB gene expression. Hence, it could be suggested that these effects refer to the dose of this flavonol glycoside, which acts as a prooxidant and does not improve the lipid peroxidation induced by T2DM in the kidney.

Glomerular damage may accelerate tubulointerstitial injury by multiple pathways such as tubular chemokine expression that results in inflammatory cell infiltration in DN (30). Abnormality in the blood rheology and increased erythrocyte accumulation are obvious in T2DM, and it may be related to excess ROS (31). Hyperglycemia in T2DM promotes apoptosis via the generation of free radicals and oxidative stress in several cell types such as glomerular and tubular cells (32). It has been revealed that myricitrin effectively reduced the number of apoptotic cardiomyocytes via inhibition of oxidative stress (33). Consistent with previous studies, present results indicated that STZ-NA-induced T2DM leads to increased RBC accumulation, inflammatory cell infiltration, apoptosis, and weight reducing in the kidney tissue, and administration of myricitrin and its SLN improved these variables. Also, these effects were more prominent in low and moderate doses of administration because these doses were more potent in the improvement of lipid peroxidation, antioxidant defense, and inflammatory gene expression.

## Conclusion

The obtained results indicated that T2DM induced by STZ-NA causes to DN, kidney apoptosis and, inflammation via the increasing lipid peroxidation and decreasing antioxidant defense. Moreover, administration of myricitrin and its SLN in low and moderated doses improved all of the DN changes through reducing oxidative stress and increasing antioxidant enzymes level, and these effects were more potent in the SLN-administered diabetic mice. 
